# High Performance Aqueous Zinc-Ion Batteries Developed by PANI Intercalation Strategy and Separator Engineering

**DOI:** 10.3390/molecules29133147

**Published:** 2024-07-02

**Authors:** Ling Deng, Kailing Sun, Jie Liu, Zeyang Li, Juexian Cao, Shijun Liao

**Affiliations:** 1 School of Physics and Optoelectronics & Hunan Institute of Advanced Sensing and Information Technology, Xiangtan University, Xiangtan 411105, China; 2 School of Energy, Mechanical and Electrical Engineering, Hunan University of Humanities, Science and Technology, Loudi 417000, China; 3 The Key Laboratory of Fuel Cell Technology of Guangdong Province, School of Chemistry and Chemical Engineering, South China University of Technology, Guangzhou 510641, China

**Keywords:** vanadium pentoxide (V_2_O_5_), aqueous zinc-ion batteries, polyaniline, Zn dendrite, separator modification

## Abstract

Aqueous zinc-ion batteries (ZIBs) have attracted burgeoning attention and emerged as prospective alternatives for scalable energy storage applications due to their unique merits such as high volumetric capacity, low cost, environmentally friendly, and reliable safety. Nevertheless, current ZIBs still suffer from some thorny issues, including low intrinsic electron conductivity, poor reversibility, zinc anode dendrites, and side reactions. Herein, conductive polyaniline (PANI) is intercalated as a pillar into the hydrated V_2_O_5_ (PAVO) to stabilize the structure of the cathode material. Meanwhile, graphene oxide (GO) was modified onto the glass fiber (GF) membrane through simple electrospinning and laser reduction methods to inhibit dendrite growth. As a result, the prepared cells present excellent electrochemical performance with enhanced specific capacity (362 mAh g^−1^ at 0.1 A g^−1^), significant rate capability (280 mAh g^−1^ at 10 A g^−1^), and admirable cycling stability (74% capacity retention after 4800 cycles at 5 A g^−1^). These findings provide key insights into the development of high-performance zinc-ion batteries.

## 1. Introduction

Ever-growing energy consumption and environmental pollution concerns seriously hinder sustainable social development. It is exigent to explore modern energy storage systems (ESSs) with the advantage of safety ecofriendly and high energy density to efficiently utilize renewable energy sources such as solar, wind, hydropower, etc. [1,2]. So far, commercial rechargeable ESSs have been dominated by lithium-ion batteries (LIBs) for their light weight, high energy density, and extended cycle life [3]. However, the limited lithium reserve, the toxic and flammable organic electrolyte, and strict water-free production conditions are intrinsic disadvantages that make them uncompetitive in coping with the upcoming challenges of large-scale energy storage [4]. Fortunately, aqueous metal-ion batteries, especially zinc-ion batteries (ZIBs), are coming into the spotlight owing to the unique merits of high energy density, low cost, environmental friendliness, and reliable safety [5,6].

The development of high-performance ZIBs is mainly hindered by the lack of suitable cathode materials with high capacity, stable structure and excellent electrical conductivity, and the formation of Zn dendrites. To date, four main categories—Mn-based compounds, V-based compounds, Prussian blue analogs, and organic compounds—have been employed as cathodes of ZIBs [7,8]. Among them, V-based oxides with fairly high capacity and good stability have been primarily studied owing to the multivalence of vanadium, layered structure, and economic efficiency. Based on the generally accepted intercalation mechanism, large layer spacing is needed to reversibly accumulate large hydrated zinc ions (0.55 nm) for the charge and discharge steps [9]. Meanwhile, some studies argue that the electrochemical H^+^ intercalation/extraction is involved in the energy storage process, enhancing the specific capacity of the cathode [10,11,12,13,14]. However, the typical layered structures of V-based oxides have suffered severe structural collapse, poor electrical conductivity, and dissolved in the faintly acid electrolyte, which is a considerable challenge for the long-cycle durability of the electrode materials. Organic molecules (PANI/PEDOT/PEO, etc.) have proven to be efficient “pillars” for stabilizing the fragile layered structure and enhanced diffusion kinetics [15]. Polyaniline is a typical conductive organic polymer based on the p-electron conjugated structure in molecular chains [16]. Recently, polyaniline has been appealing to research interests in ZIBs for its high conductivity (250 S cm^−1^) and high capacity (>100 mAh g^−1^) [17]. It is worth mentioning that the layered vanadium oxide intercalated with conductive polymer has been extensively investigated due to its specific ion charge-transport characteristics [18,19,20]. In particular, the vanadium oxide intercalated with PANI chains was demonstrated to expand the mesopores and enhance the electrical conductivity of the nano-composite. Unfortunately, to my knowledge, most of the reports have focused only on the improvement of the cathode performance of intercalated polyaniline. However, little attention is paid to its effect on the electrolyte and Zn anode. That requires more systematic research and a deeper understanding of the zinc storage mechanism.

On the other hand, the leaf-like morphology of Zn dendrite can pierce the separator and promotes side reactions between the Zn metal electrode and the electrolyte, which finally cause the batteries to fail suddenly [21,22,23]. A large number of studies have shown that the uniformity of the electric field and the ion diffusion rate can lead to the non-uniform deposition of zinc and cause dendrite growth [24,25,26,27]. Moreover, according to the Pourbaix diagram, despite the overpotential, the hydrogen evolution reaction (HER) is unavoidable during Zn deposition. Such side reaction could in turn point to the interfacial H^+^ concentration (i.e., pH) which is a critical indicator that affects Zn anode stability and reversibility [28]. The positive electrode is a major factor affecting the pH change of the electrolyte. Therefore, it is very important to study the effect of the cathode on the electrolyte and Zn anode.

Herein, the hydrate V_2_O_5_ is synthesized with PANI intercalated (PAVO_−x_) and without PANI (HVO). The cycle performance studies display that the PAVO_−2_ has a slower trend of capacity decay than HVO and PAVO_−1_. However, both PAVO_−1_ and PAVO_−2_ will suddenly terminate their charge–discharge cycles. Moreover, the high PANI contented PAVO_−2_ has a minimum of 281 cycle end laps. The ex situ pH -testing demonstrated that the high-content PANI-intercalated hydrated V_2_O_5_·3H_2_O exhibited more obvious pH variation (0.6) during charge and discharge progress. Hence, the PANI intercalation can act as a pillar to stabilize the layer structure of V_2_O_5_·3H_2_O. Meanwhile, it can also cause a greater pH change in the electrolyte, resulting in zinc anode dendrite growth. Furthermore, in situ pH investigation shows that the laser-reduced graphene modified separator can suppress pH change caused by the cathode and extend the cycle life of PAVO_−2_. The unique Zn//PAVO_−2_ cell presents excellent electrochemical performance with enhanced specific capacity (362 mAh g^−1^ at 0.1 A g^−1^), significant rate capability (280 mAh g^−1^ at 10 A g^−1^), and admirable cycling stability (maintained 74% specific capacity after 4800 cycles at 5 A g^−1^). This work presents a comprehensive understanding of the synergistic effects of PANI-intercalated cathode and GO-modified separator on the electrochemical performance of ZIBs, which further achieves a valuable design for the development of high performance ZIBs. 

## 2. Results and Discussion

**Figure 1:**
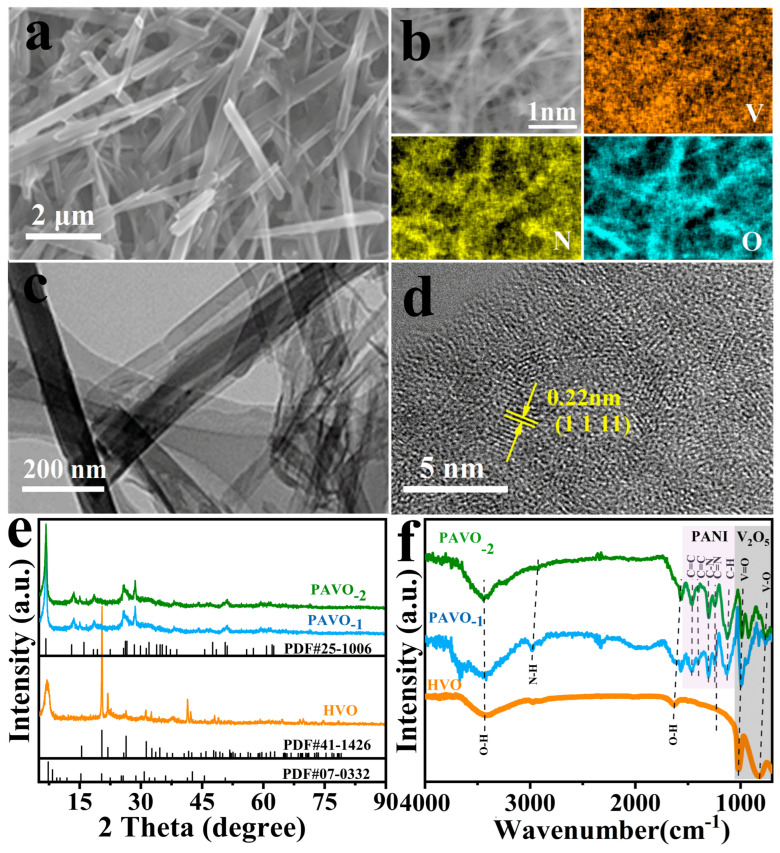
(**a**) SEM image; (**b**) SEM image and its corresponding elemental mappings of V, N, and O; (**c**) TEM image; (**d**) HRTEM image of PAVO_−2_; (**e**) XRD patterns; and (**f**) FTIR spectra of HVO, PAVO_−1_, and PAVO_−2_.

The structure and morphology of the as-synthesized PAVO and HVO were first characterized. Scanning electron microscopy (SEM), energy dispersive X-ray spectroscopy (EDS) elemental mapping and transmission electron microscope (TEM) were characterized. As displayed in Figure 1a and Figure S1b, the PAVO_−2_ and PAVO_−1_ exhibit nanobelt morphology, while the PAVO_−2_ has a uniformly large aspect ratio. In contrast, The HVO shows irregular block morphology (Figure S1a). The large aspect ratio makes PAVO_−2_ with a large specific surface area, which may be favorable for providing more active sites for zinc storage. The EDS mapping images (Figure 1b) revealed that the elements V, N, and O were homogeneously distributed in PAVO_−2_, implying the successful preparation of PANI-intercalated V_2_O_5_. The TEM image (Figure 1c) reveals the ultrathin properties of the building nanobelts. The HRTEM image (Figure 1d) presents an interplanar spacing of 0.22 nm, which is indexed to the (801) plane of V_2_O_5_·3H_2_O.

The XRD patterns of PAVO and HVO were compared in Figure 1e. The diffraction peaks for the material without PANI correspond with the theoretical main diffraction peaks of V_2_O_5_·3H_2_O (PDF No. 07-0332), demonstrating the successful synthesis of V_2_O_5_·3H_2_O crystals for all cases. The peaks beyond 50° are well matched with V_2_O_5_ (PDF No. 41-1426), indicating the V_2_O_5_ precursor does not react completely. For the materials with PANI, all peaks of PAVO_−1_ and PAVO_−2_ are indexed to V_10_O_24_·12H_2_O (PDF No. 41-1426). The peaks located at 6.6° are the 002 peaks of V_10_O_24_·12H_2_O. The space of d_002_ is ~1.4 nm which is wide than that of HVO located at 6.9° (d_001_ of V_2_O_5_·3H_2_O) Furthermore, the 002 peak of PAVO_−2_ is slightly to left of that of PAVO_−1_ which indicates the d_002_ becoming wider, accompanied by more polyaniline intercalation [29,30]. The enlarged interlayer space would effectively promote Zn^2+^ intercalation/deintercalation, therefore leading to improved electrochemical performance for PAVO [31]. The chemical structure and functional groups of PAVO and HVO were confirmed by FTIR analyses and shown in Figure 1f. The peaks located at 3421 and 1637 cm^−1^ are indexed to the hydroxyl vibration of water molecules [32]. The peaks at 831 and 1018 cm^−1^ were assigned to the stretching vibrations of O-(V)_3_ and V=O bonds, respectively [29,33]. The different stretching vibrations of V and O indicate the different structures of HVO with PAVO, which are consistent with the XRD characterization. In particular, the additional diffraction peaks of PAVO located at 1126, 1243, 1303, 1465, 1571, and 2998 cm^−1^ were ascribed to the C-H in-plane blending, C=N stretching, C-N stretching, C=C benzenoid stretching, C=C quinoid stretching, and N-H stretching, respectively, which further demonstrates the successful intercalation of PANI in the V_2_O_5_ host [29]. In addition, the composition of PAVO was further investigated by TG (Figure S2). Two stepwise weight losses occurred below 350 °C, which were attributed to the evaporation of physically adsorbed water (4.8%) on the surface of PAVO_−1_ and structural water (9.7%) in the crystals, respectively. Then, with a continuous endothermal process, there was a significant weight loss of 13%, which could be attributed to the thermal decomposition of PANI [30]. Those results were consistent with the XRD results discussed above.

The element compositions and bonding characteristics of HVO and PAVO were further evacuated by XPS. The full survey spectra of HVO and PAVO_−2_ were depicted in Figure S3 and confirmed the existence of elements V, O, and N in the samples. Compared to the HVO, the peak at ~401 eV can be observed in PAVO_−2_ corresponding to N 1s, indicating the successful intercalation of PANI [29]. As shown in Figure S4, there are two typical peaks in the energy range of 512–528 eV for HVO and PAVO_−2_, corresponding to V 2p_3/2_ and V 2p_1/2_, respectively. For HVO (Figure S4a), the peaks located at binding energies of 516.5 eV and 517.8 eV can be assigned to V^4+^ and V^5+^, respectively. While the peaks of PAVO_−2_ (Figure S4b) located at binding energies of 516.5 eV and 515.6 eV can be ascribed to V^4+^ and V^3+^, respectively, reflecting that the reduction from V^5+^ (V^4+^) to V^3+^ due to oxidative polymerization of aniline monomers to polyaniline [29]. The XPS further confirmed the successful intercalation of electrically conductive PANI into V_2_O_5_ and could facilitate the charge transport.

**Figure 2:**
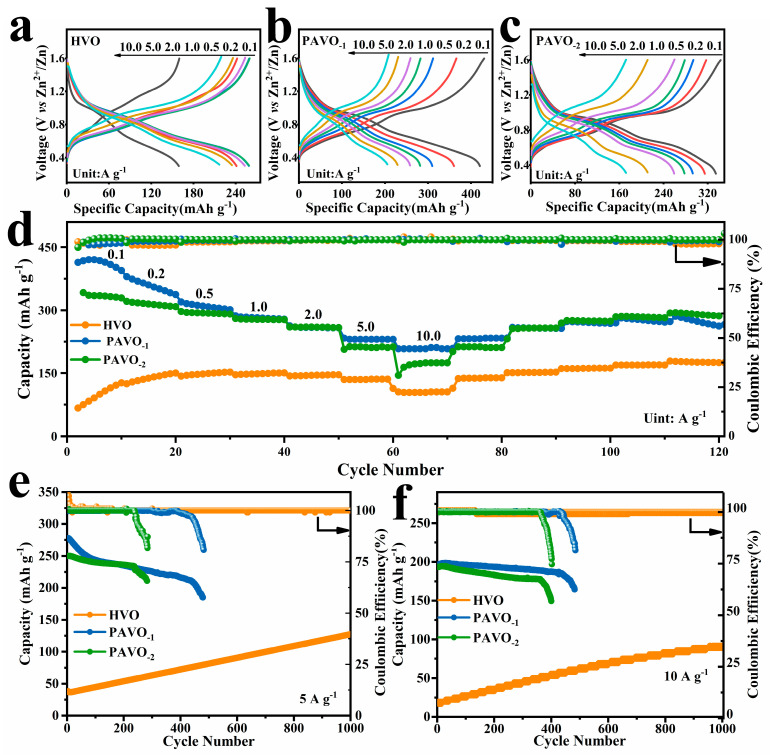
Electrochemical performance of HVO, PAVO_−1_, and PAVO_−2_ cathodes. (**a**–**c**) Charge/discharge curves at different current densities; (**d**) rate capability at various current densities; long-term cycling performance at (**e**) 5 A g^−1^ and (**f**) 10 A g^−1^.

The electrochemical properties of the as-prepared HVO and PAVO electrodes were investigated by the coin cell, in which zinc sheet was used as the anode, GF membrane as the separator and 3 M Zn(CF_3_SO_3_)_2_ aqueous solution as the electrolyte. Figure 2 illustrates the capacities of HVO and PAVO electrodes at various current densities. For PAVO_−1_, capacities of 430, 360 310 280 260, 220, and 200 mAh g^−1^ are obtained at current rates of 0.1, 0.2, 0.5, 1, 2, 5, and 10 A g^−1^, respectively, and at rates of 260, 255, 245, 240, 225, and 165 mAh g^−1^ for HVO and 340, 320, 300, 280 265, 220, and 175 mAh g^−1^ for PAVO_−2_, respectively. Even at the high current density of 10 A g^−1^, the PAVO_−1_ still delivers a capacity of 200 mAh g^−1^, indicating a preferable rate performance. The cycling performance of HVO, PAVO_−1_ and PAVO_−2_ are compared at high current densities of 5 A g^−1^ and 10 A g^−1^. As shown in Figure 2e,f, the capacities of PAVO_−1_ and PAVO_−2_ cathodes are much higher than those of HVO at 5 A g^−1^ and 10 A g^−1^. However, both PAVO_−1_ and PAVO_−2_ will suddenly terminate their charge–discharge cycles. Moreover, the high-PANI-contented PAVO_−2_ has a minimum of 281 cycle end laps (Figure 2e). The cycling life of PAVO_−1_ and PAVO_−2_ is poorer than that of HVO. There is a significant fading in coulombic efficiency for PAVO_−1_ and PAVO_−2_ after 200 cycles and the life is no more than 500 cycles. In contrast, the coulombic efficiency of HVO is almost 100% after 1000 cycles. Furthermore, the capacity of HVO increases from 32 to 141 mAh which may be due to gradual activation of the electrode material during cycling [34]. In Figure S5, it is observed that the discharge curves of PAVO_−1_ and PAVO_−2_ do not match with charge curves as cycles increased to 400, while the 2 curves are constant for HVO. It is concluded that intercalated PANI could improve the capacity, while the high PANI content is conducive to poor cycle stability and limited lifetime caused by Zn dendrites (Figure S6). Moreover, the higher the content of PANI and the more serious the dendrite (Figure S6), the faster the battery will deteriorate. Therefore, the high-PANI-content cathode PAVO_−2_ was selected to continue with the follow-up research.

**Figure 3:**
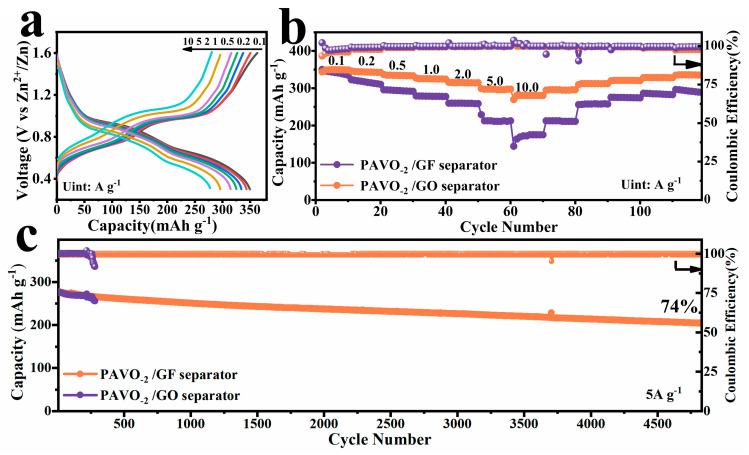
Electrochemical properties of PAVO_−2_ with GF separator or GO separator. (**a**) Charge–discharge curves of PAVO_−2_ with GO separator at different current densities; (**b**) rate capability; and (**c**) long-term cycling performance at 5 A g^−1^.

To suppress the growth of Zn dendrites, GO with unique advantages was applied to modify the GF membrane. To prepare the GO separator, the GO precursor solution were applied via electrostatic spray to the glass fibers, followed by laser irradiation. For detailed parameters, please see the experimental section. Laser irradiation can produce instantaneous local high temperatures and partially reduced the oxide grapheme [35]. As a result, the graphene oxide can attach on the glass fibers separator firmly without reunion. Figure 3b contrasts the rate performance of PAVO_−2_ with GF separator or GO separator from 0.1 to 10 A g^−1^. The capacities of the GO separator are higher than that of the GF separator. For the GO separator, the corresponding GCD curves with similar trends illustrate the capacities of 362, 350, 337, 326, 315, 297, and 280 mAh g^−1^ at current rates of 0.1, 0.2, 0.5, 1.0, 2.0, 5.0, and 10 A g^−1^, respectively. Figure 3c displays the cycling performance of PAVO_−2_ with a GF separator or GO separator. It is obvious that the GO separator still had outstanding capacity retention of 74% after 4800 long cycles at the current density of 5 A g^−1^, whereas a significant fading in coulombic efficiency was observed after only 282 cycles and finally destroyed in the GF separator. In addition, they were also evaluated in low current densities of 0.1 A g^−1^ and 0.5 A g^−1^, respectively (Figure S8). In all cases, the capacity of the GF separator decreased drastically compared to that of the GO separator, demonstrating the excellent long-term cycle stability of the GO separator. Compared to the GF membrane, the GO separator exhibits excellent electrochemical performance at both low current density and high current density.

**Figure 4:**
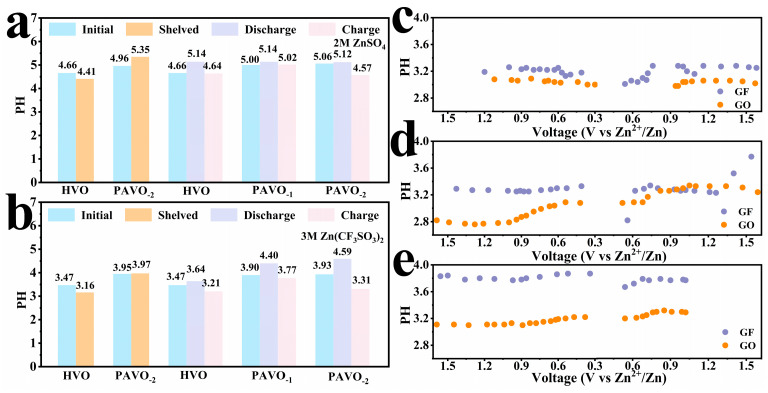
The pH of HVO, PAVO_−1_, and PAVO_−2_ cathodes under different electrolytes of (**a**) 2M ZnSO_4_; (**b**) 3M Zn(CF_3_SO_3_)_2_, in situ pH of Zn//PAVO_−2_ with GO separator or GF separator in the initial three charge/discharge cycles; (**c**) the first cycle; (**d**) the second cycle; and (**e**) the third cycle.

As we know, a high concentration of H^+^ and OH^-^ would cause the pH of electrolyte fluctuation and trigger a series of anodic side reactions [28]. The HER side reaction, which leads to Zn dendrite, is the origin of the limited lifetime of vanadium-based ZIBs [36]. Thus, the pH of cathode in 2M ZnSO_4_ and 3M Zn(CF_3_SO_3_)_2_ electrolytes with different states were investigated. As shown in Figure 4a,b, the pH evolution is generally similar in the two electrolytes. Compared to HVO, a certain increased pH value for PAVO_−2_ during shelved is observed, which may be attributed to the existence of ammonium. The EDS analysis of the GO separator after cycling further proved the existence of elemental N. As shown in Figure S9, the elemental N within the composition of PANI was detected, which shows that ammonium (NH_4_^+^) escaped from the PAVO_−2_ cathode in the electrolyte. In addition, the pH increases in discharge and decreases in charge. These results are related to the H^+^ intercalation/deintercalation mechanism in the cell [37]. More specifically, the increasing pH can result from the intercalation of H^+^ into cathode companying with Zn^2+^, HER, and oxygen reduction side reaction upon discharge. The pH decreases in charge because of the occurrence of H^+^ deintercalation from the cathode. It is concluded that the intercalation/deintercalation of H^+^ causes the pH fluctuation. Such a fluctuation could give rise to the unstable interface between Zn and the electrolyte, promoting the growth of dendrites. As a result, the dendrites caused by the uneven deposition of zinc pierced the separators and caused short circuits.

The electrochemical in situ pH of the Zn//PAVO_−2_ cell with GO separator or GF membrane was tested for the initial three cycles. Figure 4c–e illustrates the pH versus voltage trend in every cycle. In the first cycle (Figure 4c), the pH evolution of the cell with the GO separator is almost similar to that of the GF membrane. At the end of the second cycle (in charge, Figure 4d), the pH of the GF membrane ascended dramatically from 3.2 to 3.8, and it was maintained in the third cycle (Figure 4e). For the cell with the GO separator, the pH fluctuates slightly around 3.2 in the cycles, which effectively alleviates the pH fluctuation. Thus, it is concluded that the graphene oxide with functional groups such as carboxyl groups can dynamically dissociation/complex to resile against severe pH fluctuation [38]. On the other hand, the Zn dendrite growth is inhibited, which is attributed to the preferential growth of the non-protruding (002) crystal plane instead of the (101) plane and the uniform nucleation of Zn^2+^ under the action of GO [39].

**Figure 5:**
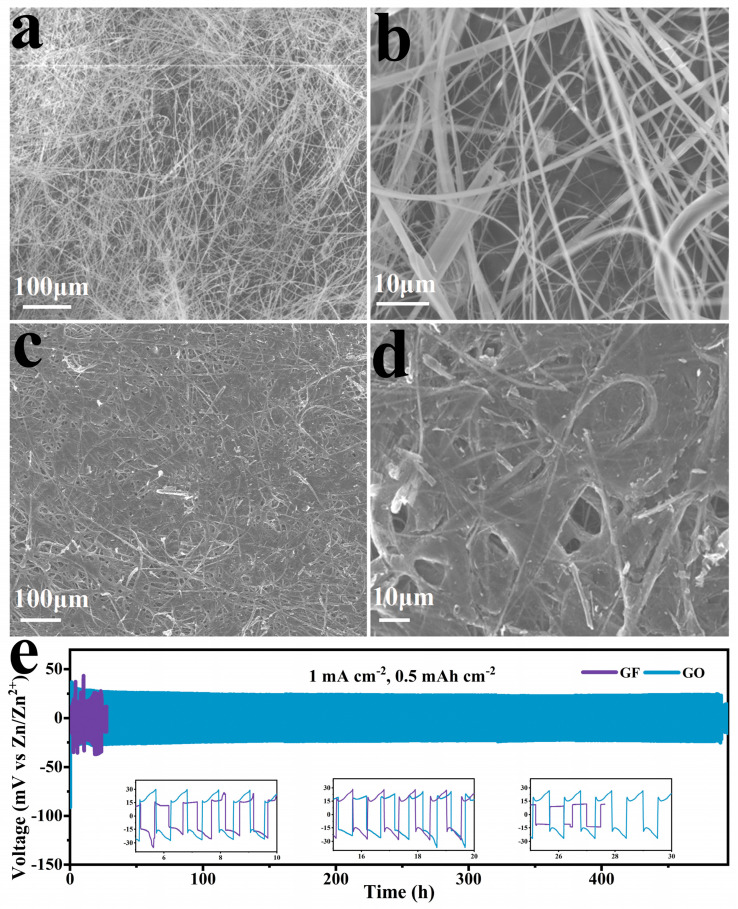
SEM of (**a**,**b**) GF membrane and (**c**,**d**) GO separator prepared with 10 mL GO suspension, (**e**) cycling performance of Zn||Zn symmetrical batteries with GO separator or GF separator at 1 mA cm^−2^ and 0.5 mAh cm^−2^. Insets are the enlarged galvanostatic voltage profiles.

The microstructures of the GF separator and GO separator were performed. In this work, different volumes of GO suspension (2, 5, 10, and 15 mL) were used in the GO modified separator and denoted as GO-2, GO-5, GO-10, and GO-15, respectively. As shown in Figure 5, the graphene film was formed on the surface of the GO separator (Figure 5c,d), while large gaps between fibers are visible in the GF membrane (Figure 5a,b). The thin film became continuous and uniform as the content of GO suspension reached 10 mL. As the content of GO suspension continued to increase, the GO on the surface of the GF membrane was agglomerated (Figure S8).

To demonstrate the potential of the optimized GO separator, we constructed ZIBs using GF membranes or GO separators and measured for the electrochemical performance. The four kinds of GO separators were assembled with a PAVO_−2_ cathode in 3M Zn(CF_3_SO_3_)_2_ electrolyte to construct ZIBs. As shown in Figure S10, the GO-10 separator possessed the best lifetime performance, so it was selected as the optimal concentration in this work and was chosen for the follow-up tests.

To investigate the effect of the GO separator on the Zn plating/stripping behavior, the Zn||Zn symmetric cells were assembled using the GO separator or GF separator. Figure 5e compares the cycling performances for Zn||Zn symmetric batteries to different separators at 1 mA cm^−2^ and 0.5 mAh cm^−2^. The GF separator was short-circuited after about 20 h. Exhilaratingly, The GO separator had a much longer cycle life of over 480 h, which was more than 20 times that of the GF separator. Moreover, the GO separator showed stable voltage during the entire Zn planting/stripping processes, whereas the GF separator exhibited rising and fluctuating voltage hysteresis from the inset pictures. It could be concluded that the novel GO modified separator can improve the cycle performance of ZIBs significantly.

**Figure 6:**
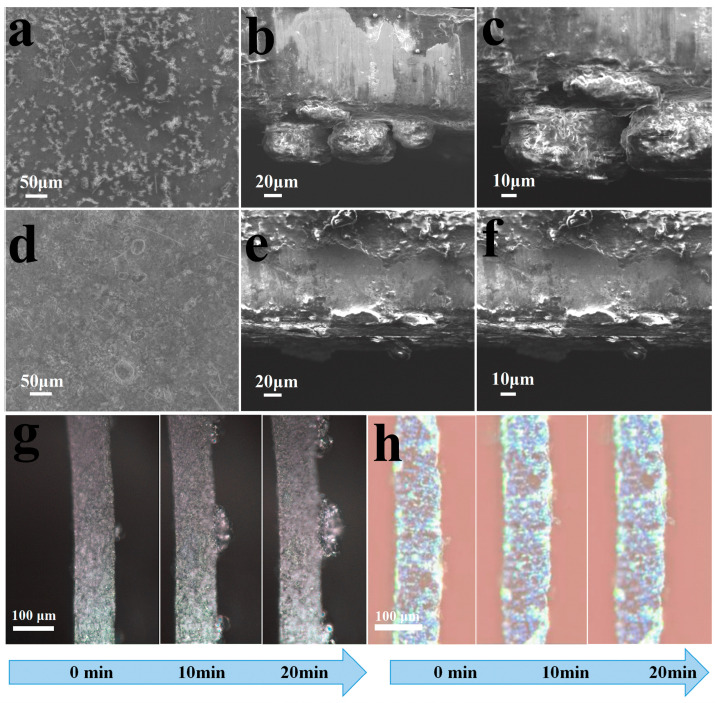
Investigation of the Zn deposition behavior. SEM images of plating/stripping morphology of Zn anode after 200 cycles at 5 A g^−1^ with (**a**) GF separator, (**b**,**c**) cross-sectional SEM images of GF separator. SEM images of plating/stripping morphology of Zn anode after 200 cycles at 5 A g^−1^ with (**d**) GO separator, (**e**,**f**) cross-sectional SEM images of the GO separator. In situ optical microscope photo of Zn plating with (**g**) GF separators and (**h**) GO separators.

To further shed light on the effects of GO film on Zn deposition behavior, the plating/stripping morphology of Zn anode after 200 cycles at a current density of 5 A g^−1^ with GO separator or GF separator in the cells (Zn sheet as anode, PAVO_−2_ electrode as cathode) were studied using SEM. For the GF separator, the zinc deposition was found to be extremely uneven with an irregular and stacked structure after 200 cycles, which could lead to the growth of Zn dendrites due to the tip effects and low reversibility for the Zn plating/stripping process, forming dead Zn [40]. As a result, Zn dendrites and dead Zn were still present on the surface of the Zn sheet after stripping due to the surficial heterogeneity of the Zn anode (Figure 6a–c). A few glass fibers were visible on the surface of the zinc anode due to dendrite penetration. Encouragingly, after 200 cycles, the morphology of the GO separator has hardly changed, and the zinc anode with the GO separator exhibited a much smooth and flat zinc layer without visible protrusions and dendrites (Figure 6d–f). Further, the in situ optical microscopy observation was conducted to identify the dynamic morphology evolution of zinc anode in the Zn||Zn cell at 1 mA cm^−1^. As shown in Figure 6g, it is readily apparent that the cells with GF separators resulted in the formation of rough and uneven Zn protrusions after only 3 min of plating time. In contrast, uniform Zn deposition was observed with the assistance of the GO separators (Figure 6h). All of those suggest the remarkable ability of GO film to regulate the Zn^2+^ distribution and suppress dendrite growth.

**Figure 7:**
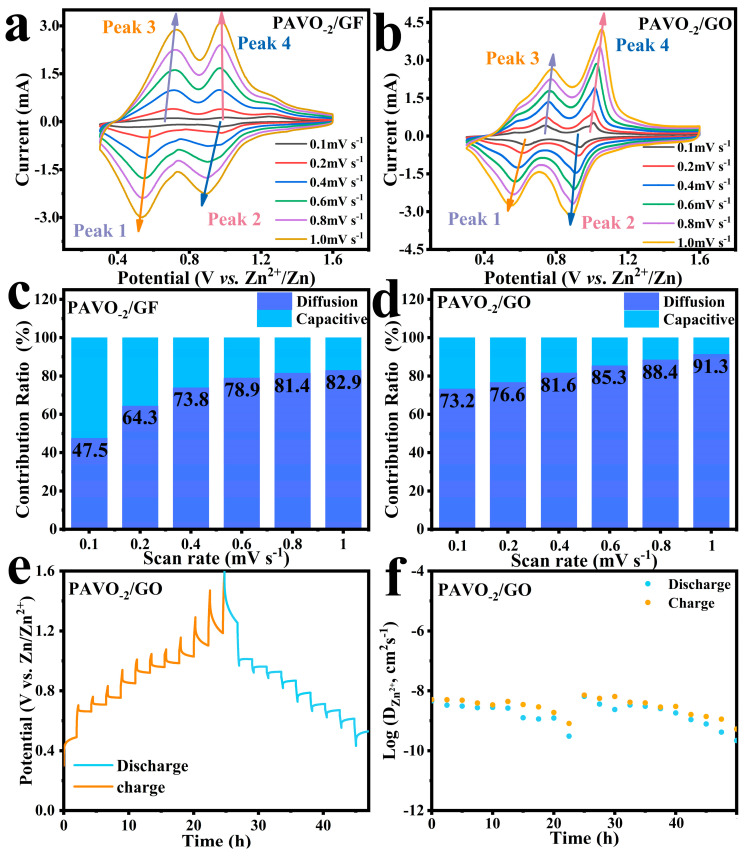
(**a**) CV curves of PAVO_−2_ with (**a**) GF separators and (**b**) GO separators at different scan rates. Percentage of pseudocapacitance contribution of PAVO_−2_ with (**c**) GF separators and (**d**) GO separators at different scan rates. (**e**) The charge–discharge curves of the PAVO_−2_ in GITT measurements and (**f**) the corresponding Zn^2+^ diffusion coefficients during the charge–discharge process.

To gain insight into the intrinsic dynamic characteristic of the cathodes with GO separator or GF separator, the cyclic voltammetry (CV) curves at various rates ranging from 0.1–1.0 mV s^−1^ were measured. As shown in Figure 7a,b, both CV curves exhibited similar redox pairs, suggesting that the GO did not affect the redox reaction of the PAVO_−2_ cathode. The peak current (*i*) increases with the rise of scanning rates (*v*), and they obey a power law relationship ($i=av^{b}$, where *a* and *b* are both adjustable parameters) [41]. The *b* values can be calculated through the test results of CV curves. For PAVO_−2_ with GF separator, the *b* values of peaks 1, 2, 3, and 4 are 1.27, 1.12, 1.43, and 1.42, respectively (Figure S11a), and those of GO separator are 0.92, 0.88, 0.87, and 0.81 (Figure S11b). Such results suggest that the electrochemical process in PAVO_−2_ has favored capacitive kinetics. Furthermore, the percentage of capacitive contribution at different scan rates was quantitatively calculated by the equation ($i=k_{1}v+k_{2}v^{1/2}$, where $k_{1}v$ and $k_{2}v^{1/2}$ represent the capacitive contribution and the ion-diffusion contribution, respectively). At a scan rate of 0.1 mV s^−1^, the capacitive contribution of PAVO_−2_ with GO separator represented by the shadow area is 73.2% (Figure S11d), which is higher than that of PAVO_−2_ with GF separator (47.5%, Figure S11c). In addition, the percentages of capacitive contribution of PAVO_−2_ with GO separators are 76.6%, 81.6%, 85.3%, 88.4%, and 91.3% at scan rates of 0.2, 0.4, 0.6, 0.8, and 1.0 V s^−1^, respectively (Figure 7d), which are higher than those of PAVO_−2_ with GF separators (64.3%, 73.8%, 78.9%, 81.4%, and 82.9%, Figure 7c), indicating that the capacitive effect plays a dominant role in the kinetics reaction and that the GO separator significantly enhances the diffusion kinetics of ions inside the PAVO_−2_ electrode, thus increasing the percentage of capacitive contribution [42].

Additionally, the Zn^2+^ diffusion coefficients (D) of different cathodes were analyzed using the galvanostatic intermittent titration technique (GITT). The corresponding GITT curve of PAVO_−2_ is shown in Figure 7e, and the values are in the range of 10^−9^ to 10^−8^ cm^2^ s^−1^ (Figure 7f). In contrast, the diffusion coefficients of HVO and PAVO_−1_ display values of approximately 10^−10^ to 10^−9^ cm^2^ s^−1^ and 10^−10^ to 10^−8^ cm^2^ s^−1^, respectively (Figure S12). This indicates that the PAVO_−2_ with GO separator shows fast ion diffusion kinetics, thus exhibiting excellent rate performance.

**Figure 8:**
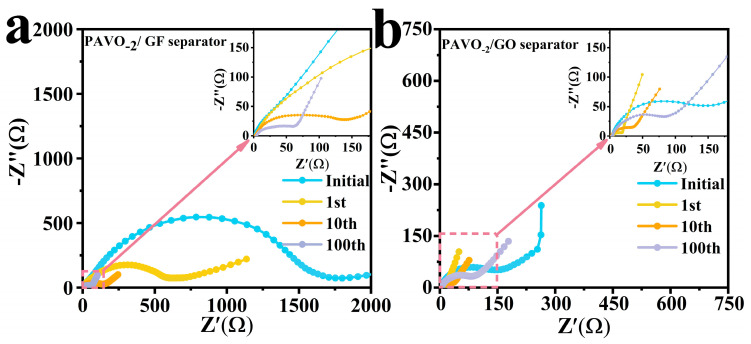
EIS spectra of PAVO_−2_ with (**a**) GF separator and (**b**) GO separator.

The EIS tests of Zn||PAVO_−2_ with GO separators or GF separators after different cycles were carried out (Figure 8). Nyquist plots are always composed of a semicircle and an oblique line. The radius of the semicircle in the first half represents the charge transfer resistance (R_ct_), which is the most important factor affecting the performance. The diagonal line in the second half corresponds to the internal resistance (R_S_) related to the ion diffusion rate. The smaller the semicircle radius and the larger the slope of the diagonal, the lower the resistance of this material [43]. According to the equivalent circuit, the initial fitting result of R_ct_ of a Zn||PAVO_−2_ cell with GO separator is ~200 ohm, which is much smaller than that of a Zn||PAVO_−2_ cell with GF separator (1000 ohm), demonstrating enhanced conductivity by GO separators. With the increase in the cycles, the resistances of all separators become smaller due to the activation of the electrodes in the cycles [35].

## 3. Experimental Section

All chemical reagents were purchased from Aladdin Industrial Co., Ltd. (Shanghai, China) and were used without further purification.

### 3.1. Synthesis of PAVO and HVO Cathode Materials

The PANI intercalated V_2_O_5_ (PAVO) was synthesized by condensation reflux in situ polymerization method. In a typical synthesis, 0.91 g V_2_O_5_ was dispersed in 100 mL deionized water stirred at room temperature for 30 min. Then, 2 mL graphene oxide (GO, prepared by Hummer’s method, 6.84 mg/mL) suspension was added into the solution under magnetic stirring. Subsequently, a given amount of aniline and 400 μL formic acid were successively added dropwise into the solution. After stirring for another 10 min, the resulting mixture was refluxed for 24 h at 100 °C under constant stirring. After cooling to room temperature, the dark green products were washed with deionized water and absolute ethanol three times by centrifugation. Finally, the PAVO materials were obtained after freeze-drying for 24 h. For comparison, PAVO_−x_ with different amounts of aniline (450 and 902 μL) were prepared and denoted as PAVO_−1_ and PAVO_−2_, respectively. To confirm the effect of PANI, the hydrated V_2_O_5_ material was synthesized in the same way without aniline and denoted as HVO.

### 3.2. Preparation of GO Modified Separator

The GF separator used is Whatman GF/A. (aperture 1.6 µm, thickness 260 µm, 150 mm diameter). The GO-modified separator was prepared by electrospinning. Generally, 10 mL GO suspension (1% quality score) was dissolved in 10 mL absolute alcohol via sonicating and vigorously stirring to get the homogeneous solution. Later, the above solution was transferred into a 30 mL syringe with a 22-gauge stainless steel needle, and the flow rate of the syringe pump was 0.05 mm min^−1^. The distance between the needle and the collector (glass fiber membrane, purchased from Whatman, Maidstone, UK) was 12 cm. When a voltage of 10.0/−4.0 KV power was supplied to the needle and the collector, the fiber was collected on the glass fiber (GF) membrane. The as-spun GO-modified separator (less than 0.1 mg/cm^−2^ GO were modified) was dried overnight at room temperature and finally reduced by a CO_2_ laser with a power of 6 W and a scan rate of 100 mm s^−1^, launched by the PLS6.150D platform (Universal Laser Systems Inc., Scottsdale, AZ, USA), and named the GO separator.

### 3.3. Materials Characterizations

Scanning electron microscopy (SEM, Carl Zeiss Gemini 500, Oberkochen, Germany) and transmission electron microscopy (TEM, (FEI, Eindhoven, The Netherlands) Titan G260-300) were applied to check the morphology and microstructure of the samples. The crystal structure of the as-obtained product was identified by an X-ray diffractor (XRD) equipped with a Bruker (Billerica, MA, USA) D8 advance (Cu Kα radiation with wavelength of 0.154 nm, scan rate of 5° min^−1^). The chemical composition of the material was detected by X-ray photoelectron spectroscopy (XPS, Thermo Scientific (Waltham, MA, USA) K-Alpha). The Thermogravimetric (TG) (PerkinElmer (Shelton, CT, USA), STA-800) was performed in the range of 30–900 °C with a heating rate of 10 °C min^−1^ under air atmosphere. The Fourier-transform infrared spectroscopy (FTIR) spectra data was collected by a Thermo Fisher (Waltham, MA, USA) Nicolet spectroscopy (NICOLET 380).

### 3.4. Electrochemical Measurements

To test the electrochemical properties of HVO and PAVO, the as-prepared materials, carbon black and PVDF were uniformly mixed in NMP with a mass ratio of 7:2:1, and then the homogeneous slurry was coated on carbon paper and dried at 60 °C for 24 h, employed as the cathodes. The mass loading of the active material was ~1.2 mg cm^−2^. The coin cells (CR2032) were assembled with the as-prepared cathode, zinc foil (100 µm in thickness, 99.999% in purity) as the anode, 3M Zn (CF_3_SO_3_)_2_ as the electrolyte, and a GF membrane or GO separator as the separator. Cyclic voltammetry (CV) was collected by a CHI-760e electrochemical workstation with scan rates from 0.1 to 1 mV s^−1^. Electrochemical impedance spectroscopy (EIS) was conducted at a frequency ranging from 0.01 HZ to 10^5^ HZ. The cycling performance, rate capability, galvanostatic charge/discharge (GCD) and galvanostatic intermittent titration technique (GITT) tests were performed in the voltage range of 0.3–1.6 V versus Zn/Zn^2+^ with a battery testing system (NEWARE, CT-4008) at room temperature.

### 3.5. pH Measurements

To measure the pH values of the battery during charge and discharge, we specially customized a mold for testing. The structure of the mold is shown in Figure S13. The cathode, diaphragm and Zn anode were sequentially placed in the pool of the base, and then electrolyte was injected. The hole on the upper cover is necessary for the probe of the pH meter to go deep into the aqueous electrolyte to measure the pH value. It is worth mentioning that the electrodes are also perforated in the center for the probe closest to the electrolyte. The pH values were measured regularly during charge and discharge.

## 4. Conclusions

In this work, we intercalated conductive polyaniline (PANI) as a pillar into the hydrated V_2_O_5_ (PAVO) to stabilize the structure of the cathode material and tested the effect of cathode on the electrolyte pH fluctuation which leads to Zn dendrite growth. Then, the laser-reduced graphene oxide modified separator was proposed to achieve stable and reversible ZIBs. The graphene oxide with functional groups such as carboxyl groups can dynamically dissociation/complex to resist severe pH fluctuation. On the other hand, it guides the preferential growth of the non-protruding (002) crystal plane instead of the (101) plane and the uniform nucleation of Zn^2+^, which efficiently inhibits Zn dendrite growth. Cooperating with the GO-modified separator, the Zn||PAVO_−2_ cell presents an excellent electrochemical performance with a high specific capacity of 362 mAh g^−1^ at 0.1 A g^−1^, a significant rate capability (280 mAh g^−1^ at 10 A g^−1^), and an excellent cycling life (maintained 74% specific capacity after 4800 cycles at 5 A g^−1^).

## Data Availability

Data are contained within the article and Supplementary Materials.

## Supplementary Materials

The following supporting information can be downloaded at: https://www.mdpi.com/article/10.3390/molecules29133147/s1, Figure S1: SEM images of (a) HVO and (b) PAVO_−1_; Figure S2: The TG curves of PAVO_−1_; Figure S3: XPS full survey spectra of HVO and PAVO_−2_; Figure S4: V 2p high-resolution XPS spectra of (a) HVO and (b) PAVO_−2_; Figure S5: Charge/discharge curves of HVO, PAVO_−1_ and PAVO_−2_ (a–c) at 5 A g^−1^. (d–f) at 10 A g^−1^; Figure S6: SEM of dendrites. (a,b) PAVO_−1_, (c,d) PAVO_−2_; Figure S7: (a) Cycle performances of PAVO_−2_ with GF separator or GO separator at (a) 0.1 A g^−1^ and (b) 0.5 A g^−1^; Figure S8: SEM of GO separator prepared with (a) 2 mL, (b) 5 mL and (c) 15 mL GO suspension; Figure S9: (a) SEM images, (b–e) the corresponding elemental mapping and (f) EDS spectrum of GO separator after several cycles; Figure S10: Cycling performance of PAVO_−2_ electrode with GO separator prepared with different GO suspensions at a current density of 5 A g^−1^; Figure S11: Log (v) vs log(i) of PAVO_−2_ with (a) GF separator and (b) GO separator plotted at peak current. CV curve of PAVO_−2_ with (c) GF and (d) GO at a scan rate of 0.1 mV s^−1^; Figure S12: The charge–discharge curves of (a) HVO and (b) PAVO_−1_ electrode in GITT measurements and the corresponding Zn^2+^ diffusion coefficients of (c) HVO and (d) PAVO_−1_; Figure S13: Structure illustration of the mold for pH tests.
