# Supplementary material for: High Performance Aqueous Zinc-Ion Batteries Developed by PANI Intercalation Strategy and Separator Engineering
Source: Molecules. 2024 Jul 2;29(13):3147. doi: 10.3390/molecules29133147 (PMC11243406; doi:10.3390/molecules29133147)
Supplement: Supplementary file 1 [file molecules-29-03147-s001.zip › molecules-2992957-supplementary.pdf]

Supporting Information

High Performance Aqueous Zinc-Ion Batteries Developed by PANI Intercalation Strategy and Separator Engineering

Ling Deng [1,2], Kailing Sun [1,*], Jie Liu [1], Zeyang Li [1], Juexian Cao [1] and Shijun Liao [3,*]

[1] School of Physics and Optoelectronics & Hunan Institute of Advanced Sensing and Information Technology, Xiangtan University, Xiangtan 411105, China
[2] School of Energy, Mechanical and Electrical Engineering, Hunan University of Humanities, Science and Technology, Loudi 417000, China
[3] The Key Laboratory of Fuel Cell Technology of Guangdong Province, School of Chemistry and Chemical Engineering, South China University of Technology, Guangzhou 510641, China
* Correspondence: skl2020@xtu.edu.cn (K.S.); chsjliao@scut.edu.cn (S.L.)

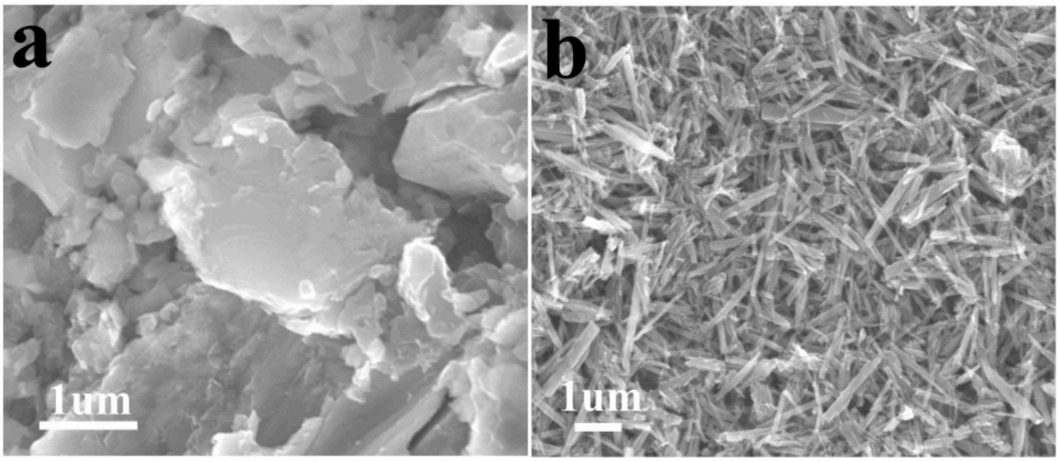

Figure S1. SEM images of a) HVO and b) $PAVO_{-1}$

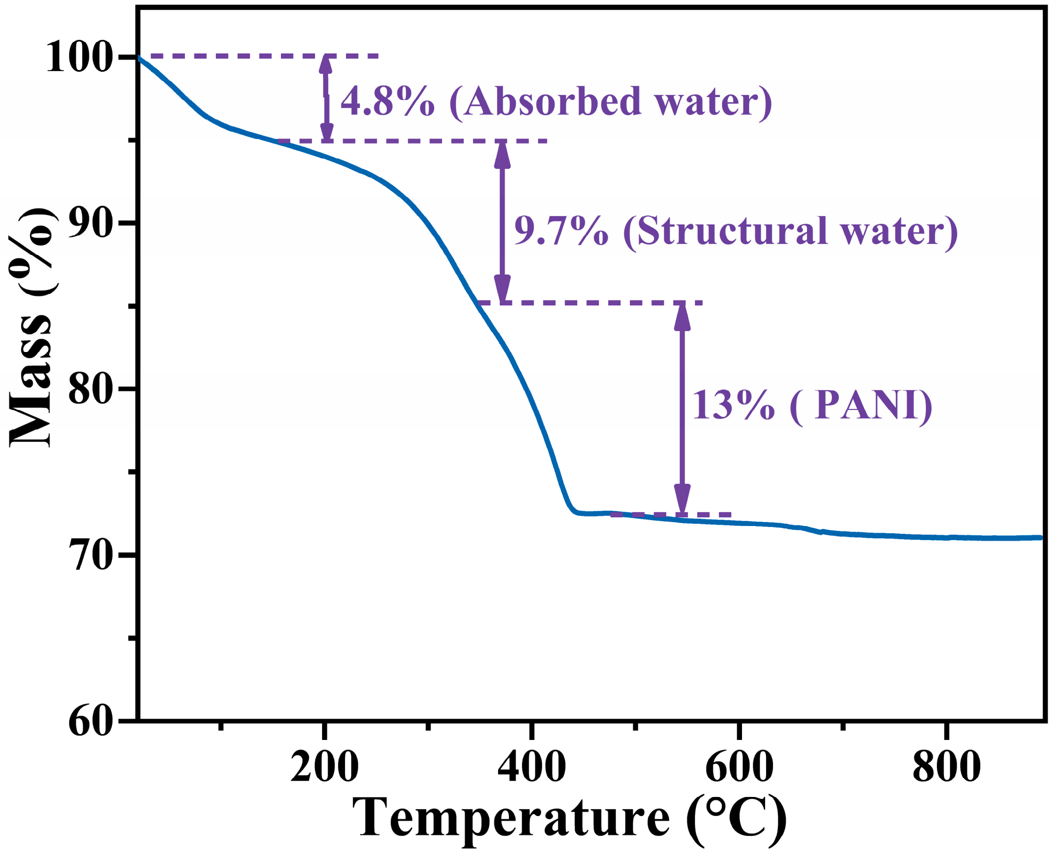

Figure S2. The TG curves of $PAVO_{-1}$.

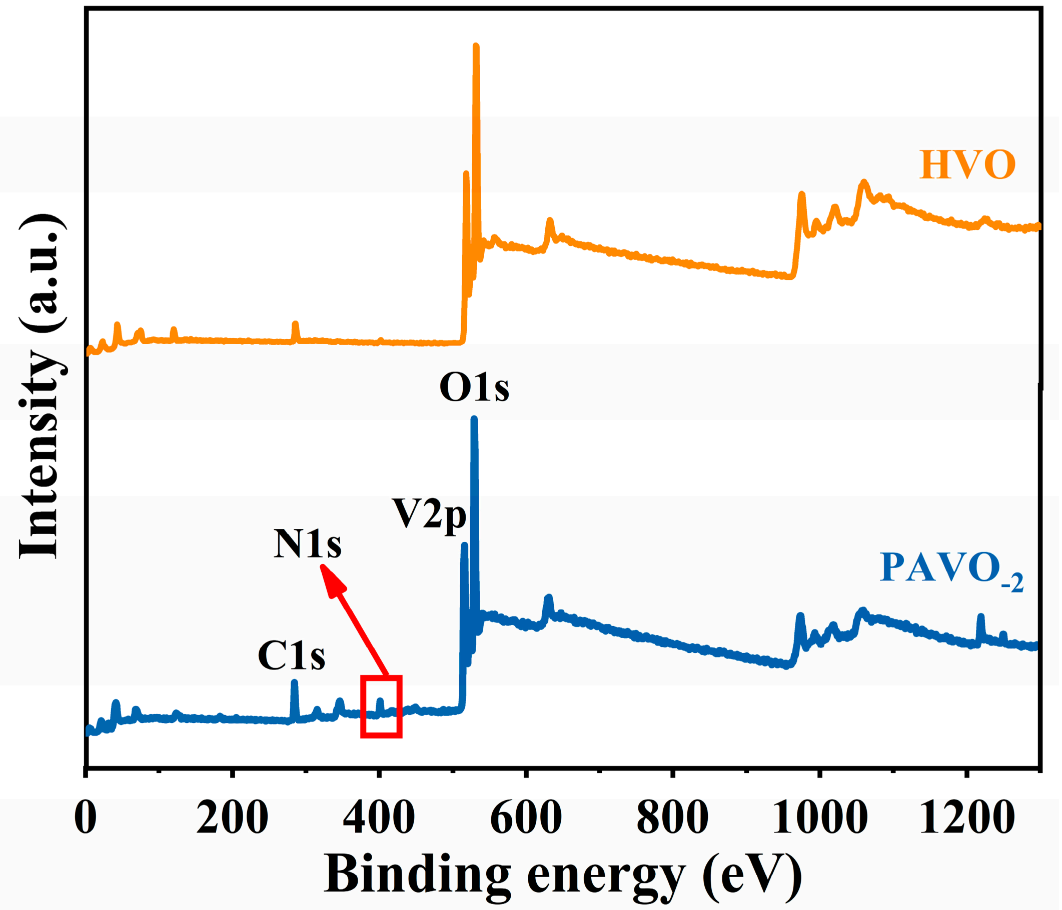

Figure S3. XPS full survey spectra of HVO and PAVO$_{-2}$

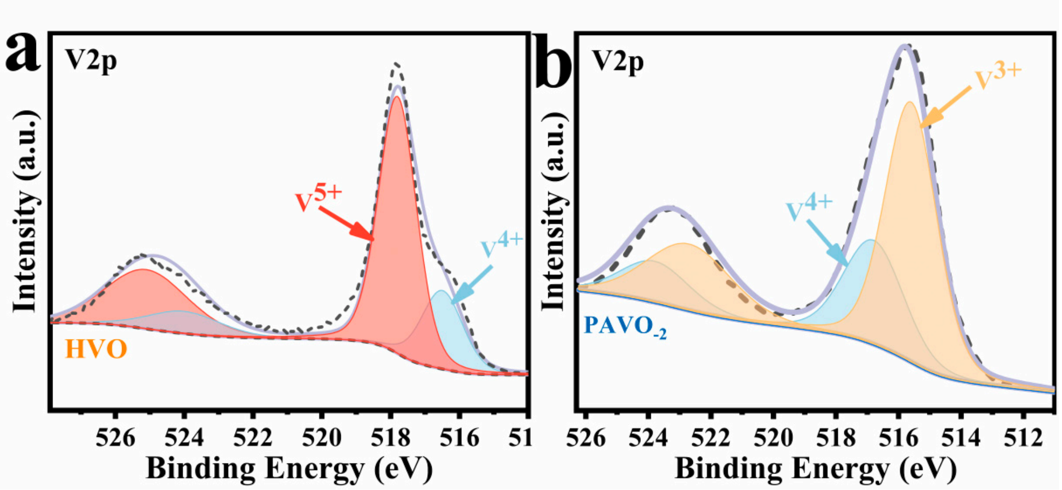

Figure S4. V 2p high-resolution XPS spectra of a) HVO and b) PAVO$_{-2}$

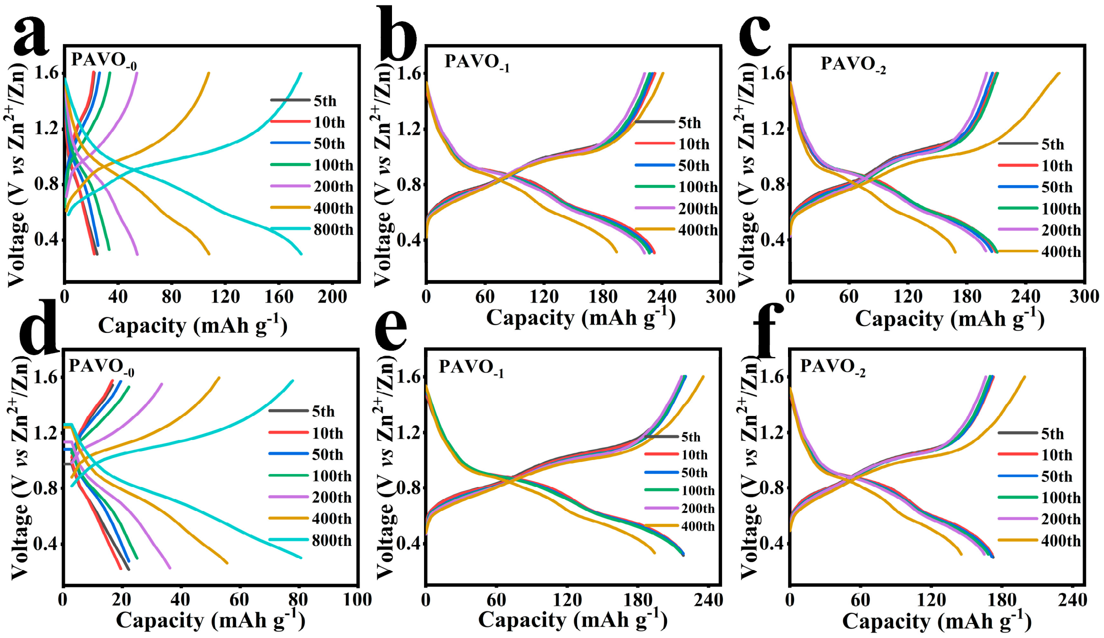

Figure S5. Charge/discharge curves of HVO, PAVO$_{-1}$ and PAVO$_{-2}$ a-c) at 5 A g^{-1}. d-f) at 10 A g^{-1}.

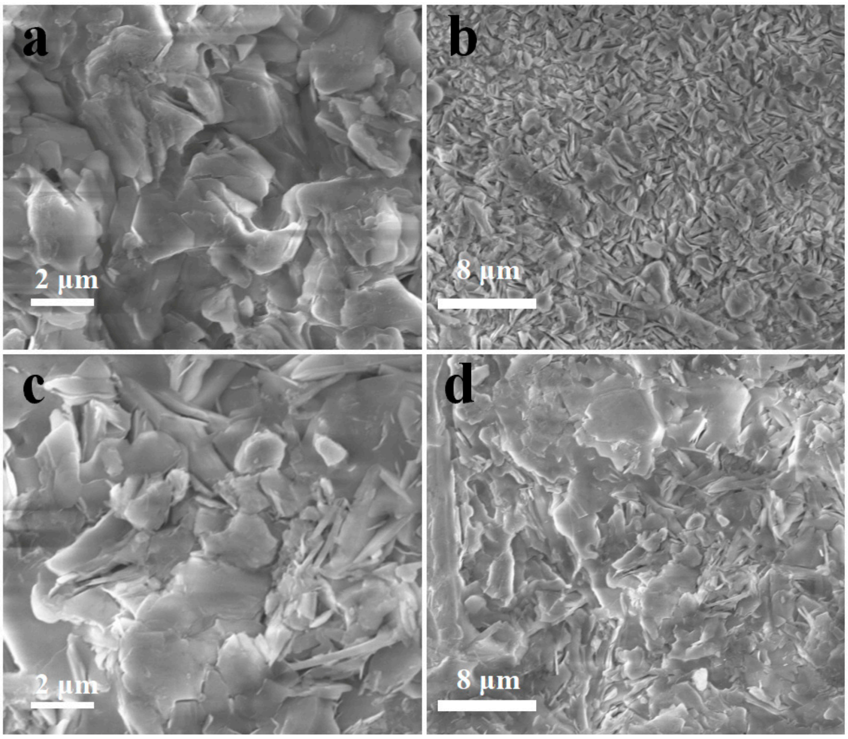

Figure S6. SEM of dendrites.a,b) PAVO$_{-1}$, c,d) PAVO$_{-2}$

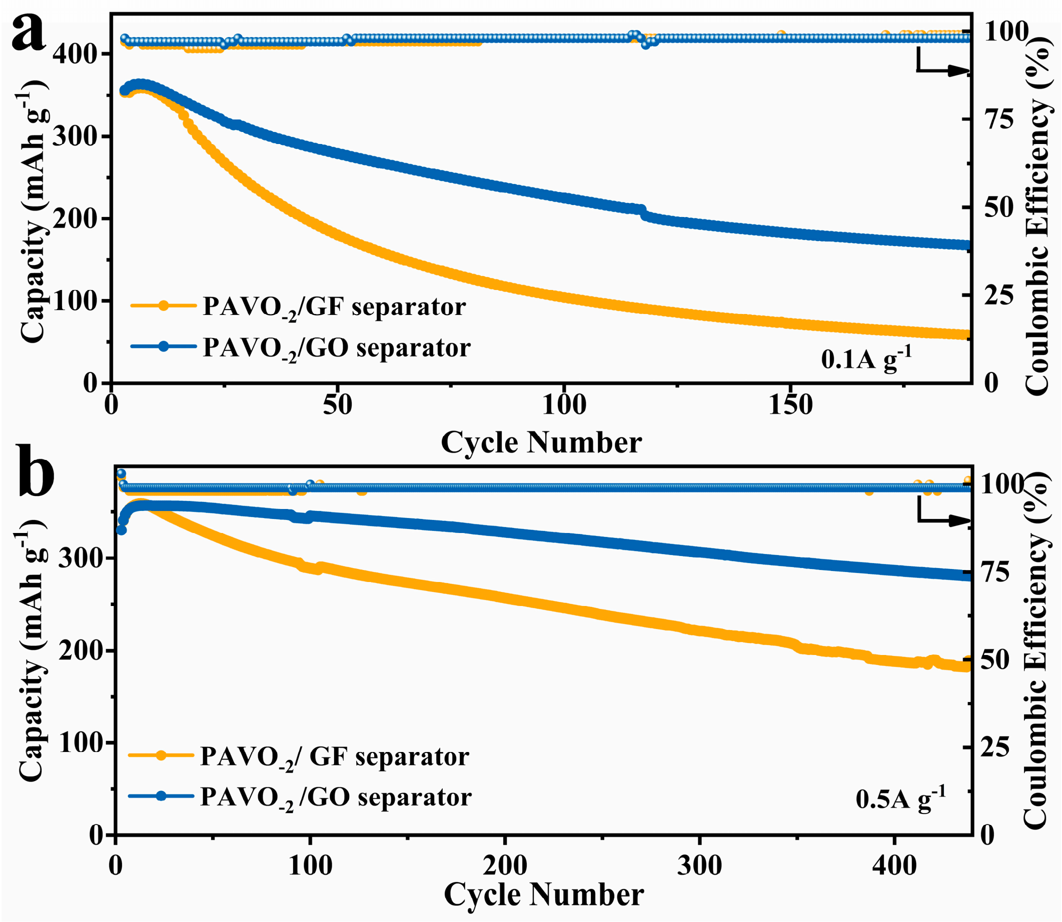

Figure S7. a) Cycle performances of PAVO-2 with GF separator or GO separator at a) 0.1 A g^{-1} and b) 0.5 A g^{-1}.

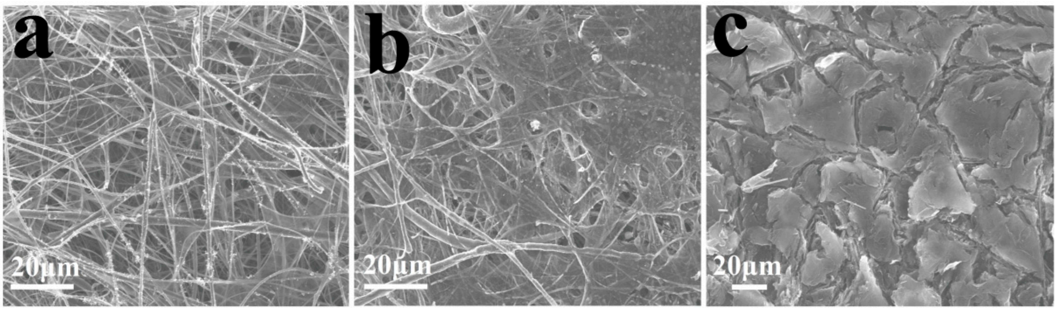

Figure S8. SEM of GO separator prepared with a) 2ml, b) 5ml and c) 15ml GO suspension.

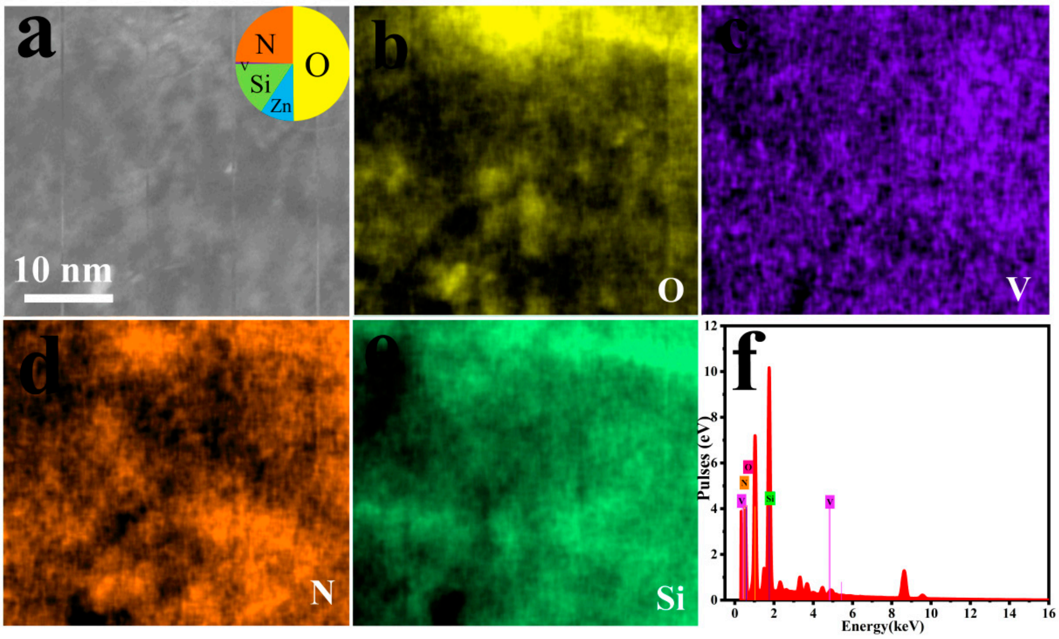

Figure S9. a) SEM images, b-e) the corresponding elemental mapping and f) EDS spectrum of GO separator after several cycles.

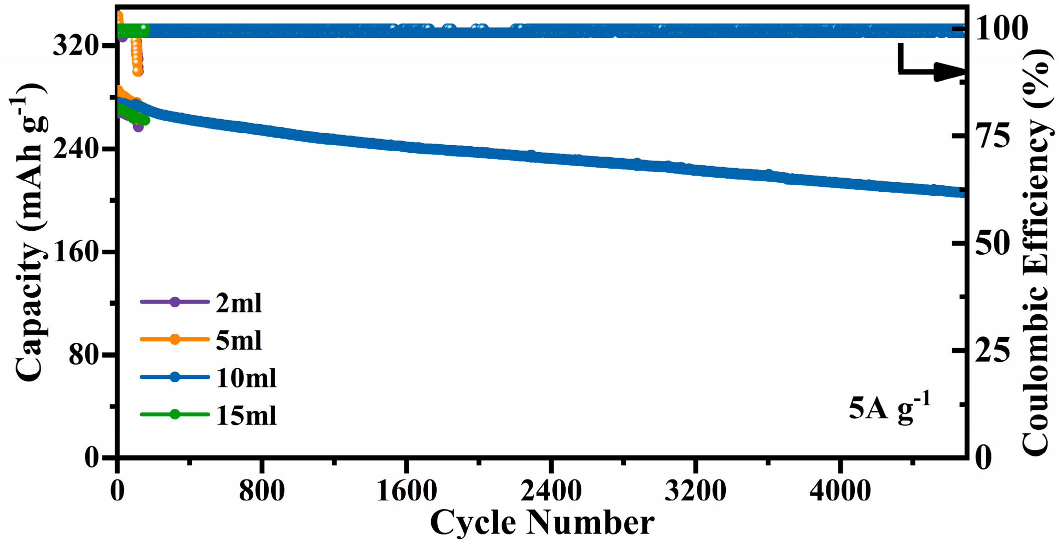

Figure S10. Cycling performance of PAVO-2 electrode with GO separator prepared with different GO suspensions at a current density of 5 A g^{-1}.

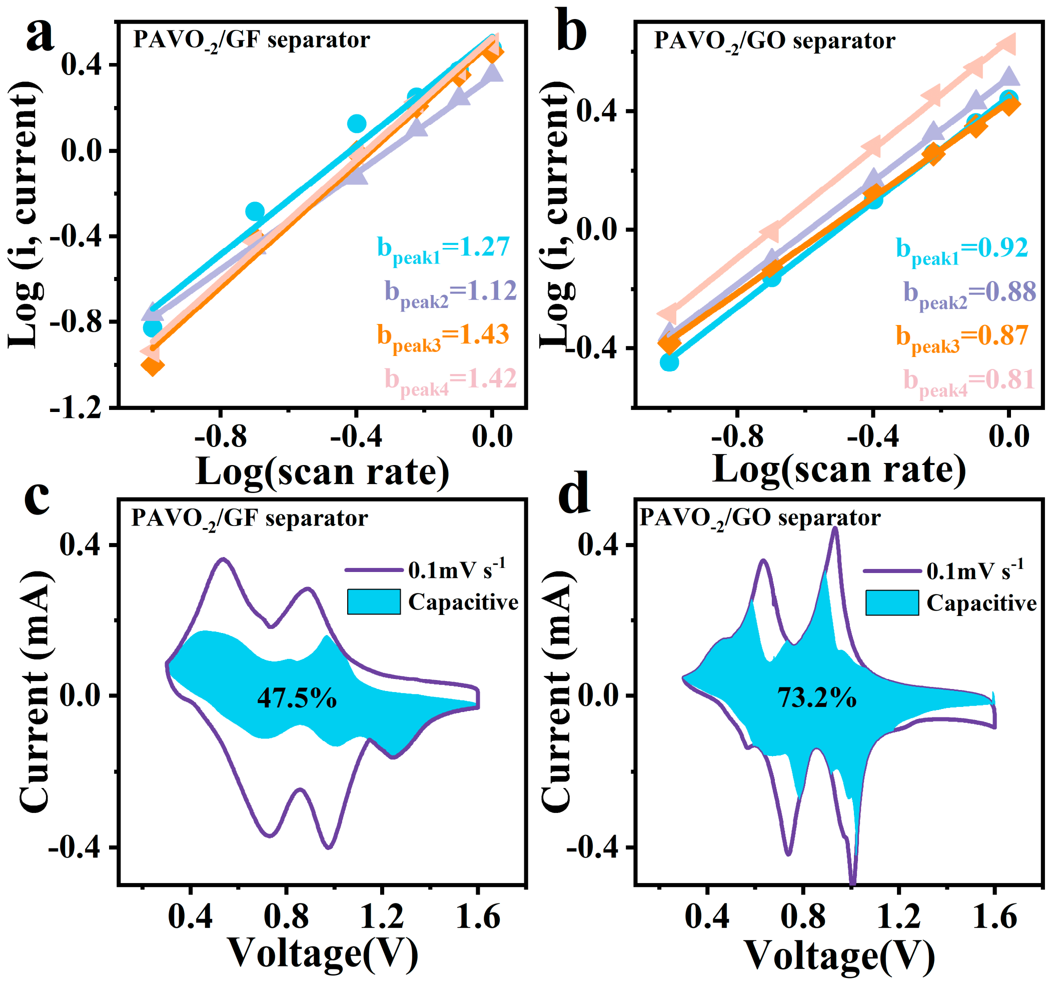

Figure S11. Log (v) vs log(i) of $PAVO_{-2}$ with a) GF separator and b) GO separator plotted at peak current. CV curve of $PAVO_{-2}$ with c) GF and d) GO at a scan rate of 0.1 mV s^{-1}.

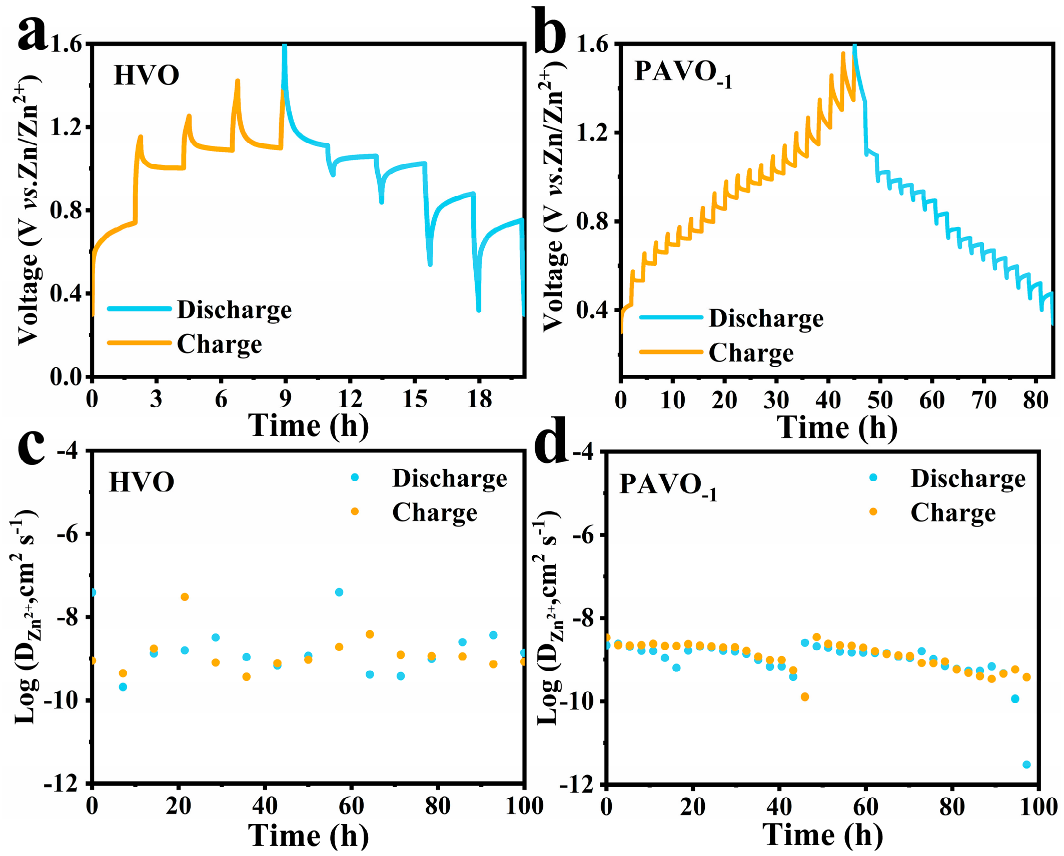

Figure S12. The charge-discharge curves of a) HVO and b) $PAVO_{-1}$ electrode in GITT measurements and the corresponding Zn^{2+} diffusion coefficients of c) HVO and d) $PAVO_{-1}$.

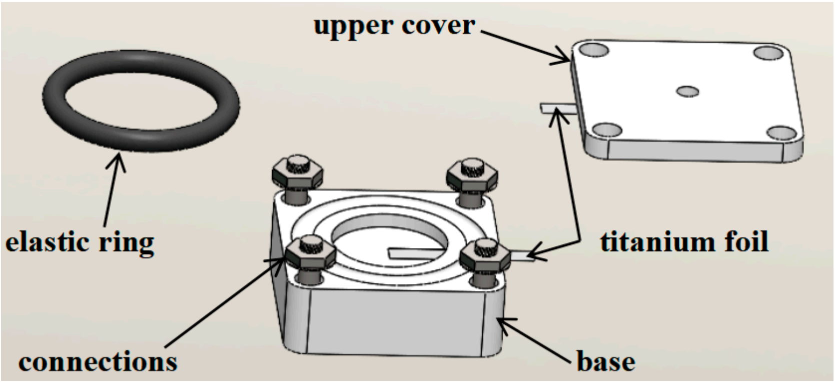

Figure S13. Structure illustration of the mold for pH tests
